# Foam Cells in Atherosclerosis: Novel Insights Into Its Origins, Consequences, and Molecular Mechanisms

**DOI:** 10.3389/fcvm.2022.845942

**Published:** 2022-04-13

**Authors:** Yuzhou Gui, Hongchao Zheng, Richard Y. Cao

**Affiliations:** ^1^Shanghai Xuhui Central Hospital, Zhongshan-Xuhui Hospital, Fudan University, Shanghai, China; ^2^Shanghai Engineering Research Center of Phase I Clinical Research and Quality Consistency Evaluation for Drugs, Shanghai, China; ^3^Department of Cardiovascular, Shanghai Xuhui Central Hospital, Zhongshan-Xuhui Hospital, Fudan University, Shanghai, China

**Keywords:** atherosclerosis, foam cell, programmed cell death, non-coding RNAs, gut microbiota

## Abstract

Foam cells play a vital role in the initiation and development of atherosclerosis. This review aims to summarize the novel insights into the origins, consequences, and molecular mechanisms of foam cells in atherosclerotic plaques. Foam cells are originated from monocytes as well as from vascular smooth muscle cells (VSMC), stem/progenitor cells, and endothelium cells. Novel technologies including lineage tracing and single-cell RNA sequencing (scRNA-seq) have revolutionized our understanding of subtypes of monocyte- and VSMC-derived foam cells. By using scRNA-seq, three main clusters including resident-like, inflammatory, and triggering receptor expressed on myeloid cells-2 (Trem2*^hi^*) are identified as the major subtypes of monocyte-derived foam cells in atherosclerotic plaques. Foam cells undergo diverse pathways of programmed cell death including apoptosis, autophagy, necroptosis, and pyroptosis, contributing to the necrotic cores of atherosclerotic plaques. The formation of foam cells is affected by cholesterol uptake, efflux, and esterification. Novel mechanisms including nuclear receptors, non-coding RNAs, and gut microbiota have been discovered and investigated. Although the heterogeneity of monocytes and the complexity of non-coding RNAs make obstacles for targeting foam cells, further in-depth research and therapeutic exploration are needed for the better management of atherosclerosis.

## Introduction

Atherosclerosis is the major cause of acute cardiovascular events including unstable angina, myocardial infarction, and ischemic stroke. It is a chronic disease of the arteries and remains asymptomatic for many years (1). Current therapies reduce the risk of cardiovascular disorders by surgical interventions and treatment of hyperlipidemia and hypertension. However, atherosclerotic cardiovascular disease (ASCVD) is still among the major cause of death worldwide. Additional therapeutic approaches are needed for reducing the risk of cardiovascular events (2).

The pathology of atherosclerosis can be divided into three stages: (a) lipid-streak stage, (b) fibrous plaque stage, and (c) advanced lesions and thrombosis (2). In the lipid-streak stage, various forms of lipids undergo retention and trapping by arterial walls. Macrophages infiltrate into the intima of the arterial wall and uptake excessive lipids, leading to the formation of foam cells (3). In the fibrous plaque stage, the vascular smooth muscle cells (VSMCs) migrate to the intima of the arterial wall and generate the fibrous cap at the atherosclerotic site. The thick fibrous cap contributes to a stable plaque. Excessive accumulation of foam cells leads to necrosis within atherosclerotic plaques (4). In the advanced lesions and thrombosis stage, the necrotic core grows and the fibrous cap diminishes. The components of the necrotic core interact with blood coagulation cells and factors and trigger thrombosis (5).

Foam cell formation is a key step in the initiation and progression of atherosclerosis (6). Lipid homeostasis is maintained by the proper function of lipid uptake, efflux, and esterification. But in the course of atherosclerosis, the excessive lipid accumulation in macrophages induces the expression of uptake receptors such as scavenger receptors (SRs) but downregulates the expression of efflux transporters like ATP-binding cassette A1 and G1 (ABCA1 and ABCG1). The dysfunction of macrophages leads to the formation of foam cells (7).

Although foam cells are the major sources of necrotic core in atherosclerotic plaques, therapies targeting foam cells are at early stages due to the limited knowledge of the foam cells-related biological mechanisms (8). In the past decades, tremendous efforts have been made on the better understanding of origins, consequences, and regulation of foam cells. Vascular smooth muscle cells (VSMC), stem/progenitor cells (SPCs), and endothelium also contribute to the formation of foam cells (9, 10). It is worth noting that monocytes can be further categorized into classical, intermediate, and non-classical subtypes in atherosclerotic plaques (11). More importantly, single-cell sequencing (sc-seq) technologies have revolutionized the knowledge of the phenotypic diversity of monocytes and VSMC-derived foam cells (12, 13). In advanced lesions, apoptosis, autophagy, necroptosis, and pyroptosis have been discovered in foam cells. Molecular mechanisms of foam cell regulation include cholesterol transporters, as well as non-coding RNAs (ncRNAs), and gut microbiota.

This review summarizes the novel insights into the origins, consequences, and regulation of foam cells in atherosclerosis, especially in the past decade. More importantly, this review identifies the obstacles to targeting foam cells. Collectively, these efforts will be helpful for further in-depth research and therapeutic exploration.

## Origins of Foam Cells in Atherosclerosis

### Monocyte-Derived Foam Cells and Their Subtypes

In atherosclerosis, monocytes are classified as classical and non-classical monocytes (14). Ly6C*^high^* monocytes in mice are categorized as classical monocytes, which are most comparable to the human CD14^++^/CD16^–^ subtype of monocytes. The classical monocytes consist of the majority of monocytes in circulation (∼90%). Ly6C*^low^* cluster of monocytes in mice is classified as non-classical monocytes, which are comparable to the human CD14^+^/CD16^++^ cluster (∼10%). The role of classical and non-classical monocytes in the progression of atherosclerosis is under debate. The classical or “proinflammatory” monocytes enter into areas of endothelial injury (15, 16). Ly6C*^high^* monocytes have migrated into the underneath of endothelium and differentiated into plaque macrophages and foam cells (17), while “non-classical” monocytes are correlated with plaque lesion size in mice. Only when the entry of Ly6*^high^* and Ly6*^low^* monocytes into sub-endothelial spaces are inhibited could atherosclerosis be ultimately halted (18). Recent studies show the existence of an intermediate subtype of monocytes in mice (Gr1*^high^*, CD43*^high^*) and humans (CD14^++^/CD16^+^) (19, 20). Higher CD14^++^/CD16^+^ subtype of monocytes in circulation is correlated with higher cardiovascular risks and events (21, 22). Compared with the classical and non-classical monocyte, the intermediate subtype of monocytes generates a great extent of inflammatory responses (23, 24). Collectively, classical, non-classical and intermediate subsets of monocytes exert effects on lipid accumulation and inflammatory response in atherosclerosis.

Macrophages, differentiated and matured from monocytes, are the central cells in atherosclerosis (25). Since the 1960s, macrophages are classified in M1 and M2 subtypes (26). It has been extensively accepted that M1 macrophages trigger and participate in the inflammation process while M2 macrophages are anti-inflammatory (27, 28). However, this classification oversimplifies the function of macrophages in atherosclerosis (29). Recent studies have challenged the established classification (27). The classification of M1 and M2 macrophages are extreme states of functions and under different stimuli, the M2 phenotype is not always athero-protective (30, 31).

In the past decade, macrophage heterogeneity has been discovered and demonstrated in the atherosclerotic plaques (11). Single-cell technologies allow accurate measurement of phenotypes of an individual cell. These advanced technologies have greatly improved the understanding of macrophage phenotype diversity. Single-cell RNA sequencing has been utilized to examine macrophage heterogeneity in multiple mouse models of atherosclerosis (32, 33) and human carotid atherosclerosis (12). More importantly, single-cell technologies including single-cell RNA sequencing, single-cell transcriptomics, make it possible for monitoring the dynamic changes of macrophage phenotypes in atherosclerotic plaques. Three main clusters of the macrophages, which are resident-like, inflammatory, and triggering receptor expressed on myeloid cells-2 (TREM2*^hi^*), have been identified (34, 35). The resident-like macrophages can infiltrate into the plaques and their phenotypes are similar to M2 macrophages. The inflammatory macrophages are non-foamy subtypes and located in the intima of the plaques. They are the major population expressing inflammatory cytokines (36). TREM2*^hi^* macrophages, the third subtype, are foamy lipid-laden macrophages, with impaired cholesterol efflux capacity, and cellular catabolic processes, which have an M2-like phenotype (32).

### Vascular Smooth Muscle Cell-Derived Foam Cells

VSMCs are flexible, non-autonomous cells that are widely distributed in multiple tissues such as blood vessels, the trachea, and the digestive tract (37). VSMCs are not terminally differentiated cells. Phenotype switching occurs under a variety of physiological and pathological conditions (9). The markers of smooth muscle cells, such as actin alpha 2 (ACTA2), myosin heavy chain 11 (Myh11), are inhibited while the markers of terminally differentiated cells are elevated, which is accompanied by the transformation of cell function. In adipose differentiation medium, VSMC undergo phenotypic transformation into adipocytes, and the expression of markers such as CCAATenhancer-binding proteins (C/EBPα), peroxisome proliferator-activated receptor-gamma (PPARγ), and leptin on the cell surface will increase (38, 39). In the state of tissue damage, the expression of SMemb/non-muscle MHC isoform-B and CRBP-1 in VSMC increases (40). In osteoporosis, VSMC can be transformed into osteoblast-like cells, with the induction of markers such as runt-related transcription factor 2 (Runx2), SRY-Box 9 (Sox9), etc. (41, 42).

In atherosclerosis, VSMC is firstly identified in the fibrous cap by using immunofluorescence. The fibrous cap is mainly composed of VSMC and its secreted collagen, elastin, etc. (43). The smooth muscle cell markers ACTA2 and SM myosin heavy chain (MHC) are highly expressed. The foam cells in the necrotic core are mainly composed of macrophages with high expression of macrophage surface markers such as Mac2 and CD68 (44). Recent studies greatly expand the origin of foam cells in atherosclerotic plaque.

Lineage-tracing technology greatly expands the understanding of the origins of foam cells in the plaques. VSMCs undergo trans-differentiation and become macrophage-like cells during atherogenesis (37). A lineage-tracing system, induced by myh11 promoter, is developed in the apoE^–/–^ mouse model of atherosclerosis. The VSMC derived foam cells in advanced atherosclerotic plaque are underestimated and a large number of VSMCs undergo phenotypic conversion with the loss of contractile markers in the plaques (45). This phenomenon of VSMC phenotype switching has also been verified in humans (46). Approximately 40% of the macrophages in the plaque are derived from VSMC, which is the product of VSMC phenotypic transformation (46). In the process of phenotypic transformation, the expression of VSMC markers is reduced or even lost. In mouse models, VSMCs, identified by traditional immunostaining, only account for about 20% of fluorescent protein-labeled VSMCs, indicating that traditional immunostaining may miss a large portion of VSMC-derived cells, thereby underestimating the VSMCs in plaques (47). The impact of transformation on disease has shown that VSMCs are an important source of plaque macrophages, and phenotypic transformation can transform VSMCs into macrophages to obtain the surface markers and functions of macrophages (48). Therefore, inhibiting the phenotypic transformation of VSMCs is beneficial to inhibit the number of macrophages in the plaque and maintain the number of VSMC in the fibrous cap, which is a potential new strategy to stabilize plaque (49). *In vitro* studies have confirmed that under the action of a variety of lipids and inflammatory stimuli, such as cholesterol (50), oxidized phospholipids (oxPL) (51), platelet-derived growth factor (PDGF) (52), VSMCs undergo the phenotypic transformation and transform into macrophage-like cells. The level of surface markers of macrophages, including CD68 and macrophage-2 (Mac2), is increased, while the expression of smooth muscle cell surface markers is decreased (53). Besides, krüppel-like factor 4 (KLF4) promotes the phenotypic switching of VSMC to macrophage-like proinflammatory cells and the formation of atherosclerotic plaques. A significant reduction in lesional size is observed in VSMC-specific knockout of KLF4 in apoE^–/–^ mice (47).

Sc-seq is a powerful and effective way to accurately identify VSMC subsets in atherosclerotic plaques. A couple of research groups have combined sc-seq with lineage tracing technology. In human atherosclerotic plaques, VSMC can differentiate into fibroblast-like cells instead of macrophages as previously reported (54). VSMC-specific knockout of Transcription Factor 21 (TCF21) inhibits phenotypic modulation in mice. TCF21 expression is strongly associated with VSMC phenotypic modulation and cardiovascular risks in humans. A recent scRNA-seq study discovers an intermediate cell state of VSMC in atherosclerotic plaques termed “SEM” cells. It can be identified in human coronary and carotid arteries with plaques. SEM undergoes differentiation into macrophage and fibrochondrocyte-like cells (13, 55).

### Other Origins of Foam Cells

**FIGURE 1:**
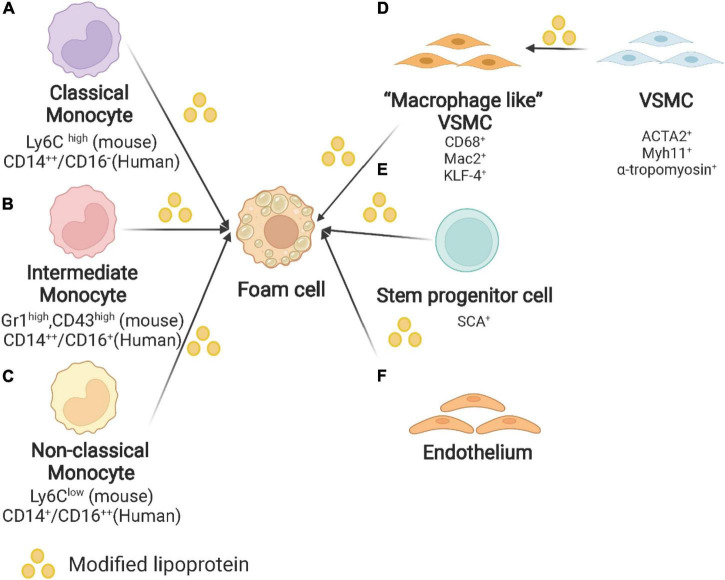
Origin of foam cells in atherosclerotic plaques. **(A)** Classical monocyte: Ly6C*^high^* monocytes in mice and CD14^++^/CD16^–^ in humans. **(B)** Intermediate monocyte: Gr1*^high^*, CD43*^high^* in mouse and CD14^++^/CD16^+^ in human. **(C)** Non-classical monocyte: Ly6C*^low^* monocytes in mice and CD14^+^/CD16^++^ in humans. **(D)** VSMC: VSMC undergoes phenotypic switching to macrophage-like VSMC and finally converts into foam cells. **(E)** Other origins of foam cells: stem/progenitor cells (SPCs) and endothelium. Created with BioRender.com.

SPCs and endothelial cells also lead to the formation of foam cells in atherosclerotic plaques (56). The reason why SPCs should be studied for atherosclerosis is that SPCs expressing stem cell antigen-1 (Sca1^+^) increase the areas of necrotic cores which consist of foam cells (57). A lineage-tracing study gives more evidence that VSMC derived Sca-1^+^ cells are capable of generating several cell types including macrophages (58). As maintaining VSMC phenotype is more atheroprotective, kruppel-like factor 4 (KLF-4) may be key in modulating this cell trans-differentiation (59). Endothelial cells have been reported to influence the entry of lipoproteins, but their contribution to foam cells is less discussed (60). The origin of foam cells in atherosclerotic plaques is depicted in Figure 1.

## Consequences of Foam Cells in Atherosclerosis

The foam cells, derived from macrophages or VSMCs, impairs efferocytosis and triggers multiple pathways of programmed cell death including apoptosis, autophagy, necroptosis, and pyroptosis. The increasing dead cells enlarge the necrotic cores in the atherosclerotic plaques and reduce the plaque stability.

### Apoptosis

The lipid overload by macrophages and VSMCs induces foam cell formation and triggers apoptosis in atherosclerotic plaques (61). Numerous papers have shown that oxLDL can initiate and activate the apoptotic pathway through the caspase cascade (62–64). The cytochrome c, a mitochondrial intermembrane protein, is delivered into the cytosol and induces the expression of apoptotic protease activating factor 1 (APAF1) and apoptosome, which triggers apoptosis cascade by activating the serine protease caspase 9. Active caspase 9 stimulates caspase 3 and caspase 7, which results in DNA fragmentation (65).

However, previous attempts to reduce apoptosis of macrophages may not lead to beneficial effects in atherosclerotic plaques. Inhibition of B-cell lymphoma-2 (Bcl-2) associated X protein (Bax) in macrophages suppresses apoptosis in macrophages but accelerates atherosclerosis (62, 66). Interestingly, inhibition of Bcl-2 leads to excessive apoptosis which induces larger necrotic areas in atherosclerotic plaques of apoE^–/–^ mice (67). Scientists have explored the reason for this diverse effect of inhibition and induction of apoptosis. In-depth studies reveal that apart from apoptosis, the ability to clearance of apoptotic cells is vital for its consequences.

Efferocytosis is an intracellular process that can resolve inflammation and prevent excessive apoptosis (68). Excessive apoptosis triggers large-scale and irreversible necrosis in plaques. Instead, with the proper function of efferocytosis, apoptotic cells are appropriately cleared (69). The accumulation of uncleared apoptotic cells triggers necrosis and releases pro-inflammatory mediators when efferocytosis capacity is impaired (70, 71). The impaired function of efferocytosis leads to a large necrotic core and unstable plaques due to insufficient clearance of dying macrophages. Efforts have been made to regulate apoptotic cells to promote efficient clearance to alleviate atherosclerosis (72). ATP citrate lyase deficiency stabilizes atherosclerotic plaque *via* enhanced apoptosis and improved efferocytosis (73). Circadian miR21 expression induces a diurnal rhythm of apoptosis with impaired function of efferocytosis and increases the size of the necrotic core (74). In early lesions, efferocytosis induces anti-inflammatory pathways and prevents cellular necrosis by activation of NF-κB signaling (75). In foam cells, Inflammatory pathways are induced by endoplasmic reticulum stress (ER stress). It promotes apoptosis by inhibiting NF-κB signaling and induction of the c-Jun N-terminal kinase (JNK) pathway (76).

### Autophagy

Autophagy is a conserved pathway for protein lysosome degradation (77). Autophagy is initiated by the formation of autophagosomes, characterized by double-membrane vesicles (78). The membrane elongation in the process of autophagosome formation is mediated by the microtubule-associated protein 1A/1B light chain 3 (LC3) conjugation system. The autophagosome is then subjected to lysosomal degradation. Unlike apoptosis, autophagy is an intracellular process for the reuse of degraded proteins. Autophagy also removes abnormal organelles and proteins for cellular homeostasis. However, impaired clearance of autophagosome triggers autophagic cell death. Autophagy abnormality participates in various diseases including cardiovascular diseases, cancer, and aging-related diseases (79). The role of autophagy in atherosclerosis is under debate. Several studies demonstrate the athero-protective effect by maintaining basal autophagy in atherosclerosis (80). Impaired autophagy abolishes the clearance of dysfunctional organelles and results in the accumulation of cytotoxic aggregates (81). Rescue of impaired autophagy by rapamycin, a mechanistic target of rapamycin (mTOR) kinase inhibitor, shows the potential for maintaining the stability of atherosclerotic plaque (82). However, excessive autophagy may induce cell death. Multiple research works demonstrate that oxidized lipids and inflammation-induced autophagy hyper-activation lead to damage to the vascular and progression of atherosclerosis (83–85).

In foam cells, autophagy contributes to the reversal of normal lipid homeostasis. It participates in the transport of lipid droplets to lysosomes for cholesterol ester hydrolysis. Autophagy involves the delivery of lipid droplets to lysosomes for hydrolysis by lysosomal acid lipase (LPL). In foam cells, intracellular free cholesterol is then subject to ABCA1-dependent efflux (86). Autophagy-related 5 (Atg5) is a key mediator of membrane elongation. The deficiency of Atg5 promotes plaque necrosis. Atg5-knockout in macrophage induces expression of p62 and down-regulated the LC3, thus the autophagy function is abolished. Furthermore, Atg5 knockout in macrophages may result in increased plaques by activation of inflammasome (87). Another study reveals that phagocyte autophagy is evoked by Akt inhibitors, mTOR inhibitors, and mTOR-siRNA, which indicates that activating autophagy of phagocytes will stabilize vulnerable coronary-artery disease plaques (87).

Collectively, these results point toward the necessity of basal level of autophagy and its protective role in atherosclerosis. However, when autophagy participates in the destruction of the cell, it exacerbates the inflammation and accelerates cell death. Future studies are necessary to explore the way for maintaining basal autophagy while avoiding excessive autophagy in atherosclerosis.

### Necroptosis

Necroptosis, which is also termed “programmed necrosis,” is an emerging pathway of programmed cell death (88, 89). It is triggered by various cell-surface receptors, including tumor necrosis factor –α (TNF-α), interferon (IFN), toll-like receptors (90). Although morphologically similar to necrosis, necroptosis is mediated by receptor-interacting serine/threonine-protein kinase 1 and 3 (RIPK1&3) (91). After the addition of caspase inhibitor zVAD, it could not only inhibit the apoptotic caspase cascade but also initiate the necroptosis pathway (92). TNF-α and TNF receptors interactions lead to three unique complexes (93). The RIP homotypic interaction motif (RHIM) induces the process of necroptosis by strengthening the interaction of RIPK3 and RIPK1 (94). RIPK1 and RIPK3 are activated by phosphorylation and sequentially phosphorylates the mixed lineage kinase domain-like protein (MLKL). This process leads to the assembly of the complex called necrosome, which is a critical step for the progression of necroptosis in humans (90). Necroptosis participates in the death of necrotic cells *via* RIPK1 and RIPK3, which is different from necrosis (91). Necroptosis has been discovered in many diseases, including cancer, cardiovascular diseases, central nerve system (CNS) syndromes, and other systematic inflammatory diseases (95).

In both early and advanced atherosclerotic plaques, oxLDL and oxPL induce macrophages to undergo RIPK1/RIPK3/MLKL mediated necroptosis (96). A couple of studies discover that RIPK1 is abundant in early lesions of atherosclerosis in humans and mice (97–99). Antisense oligonucleotides (ASO) of RIPK1 significantly attenuates lesion areas in the aortic sinus and foam cell-rich lesions are also reduced (100). Besides, in macrophages, loss of RIPK1 reduces the production of interleukins and other proinflammatory cytokines (93). However, different studies generate diverse results. RIPK1 inhibition generates different results in the early and advanced stages of atherosclerosis. RIPK1 inhibitor attenuates atherosclerotic plaques after 2-weeks of treatment, with a lower level of inflammatory cytokines. Long-term administration (4-week) of RIPK1 inhibitor accelerates atherosclerosis with increased lesion area in the plaques and more lipid accumulation in macrophages (101). By suppressing necroptosis cell death, RIPK1 inhibitor promotes foam cell formation in isolated macrophages with incubation of oxLDL (102). An in-depth investigation reveals that MLKL knockdown attenuates necroptosis and inhibits the areas of necrotic cores in the plaques. However, the total lesion area is not altered (103). Besides, treatment with MLKL antisense oligonucleotide reduces circulating cholesterol levels compared with control antisense oligonucleotide but increases the accumulation of lipids within the plaque and in macrophage foam cells (103).

### Pyroptosis

Pyroptosis is a highly inflammatory form of programmed necrosis which is initially identified in infections of bacterial and other pathogens (104). In the past decade, extensive studies have shown that pyroptosis is initiated by various kinds of inflammatory stimuli including lipopolysaccharides (LPS), oxLDL, reactive oxygen species (ROS). These stimuli activate the NF-κB pathway and the NOD-like receptor 3 (NLRP3) inflammasome. NLRP3 then causes pyroptosis through caspase-1- and caspase-11-mediated cytoplasmic protein gasdermin D (GSDMD). Caspase -1 and -11 cleave and activate GSDMD and GSDMD pores in the plasma membrane and consequently promotes pyroptotic cell death. Caspase-3 induces pyroptosis *via* cleavage of GSDME and granzyme A can cleave GSDMB to cause cell death (105).

**FIGURE 2:**
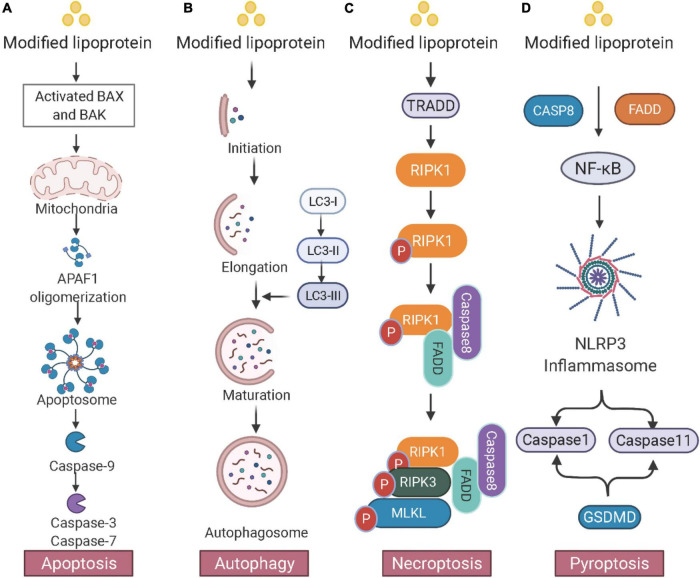
Consequences of foam cells in atherosclerosis. **(A)** Apoptosis: The mitochondrial intermembrane protein is released and triggers apoptotic protease activating factor 1 (APAF1) and apoptosome, leading to DNA fragmentation. **(B)** Autophagy: Double membrane autophagosomes are fused with lysosomes for degradation. The microtubule-associated protein 1A/1B light chain 3 (LC3) conjugation system participates in the membrane elongation and formation of the autophagosome. **(C)** Necroptosis: Activated TRADD (TNFRSF1A associated *via* death domain) triggers the phosphorylation of RIPK1 and RIPK3 and induces the downstream MLKL. **(D)** Pyroptosis: Inflammatory stimuli activate the NF-κB pathway and the NOD-like receptor 3 (NLRP3) inflammasome. NLRP3 then causes pyroptosis through caspase-1- and caspase-11-mediated cytoplasmic protein gasdermin D (GSDMD). Created with BioRender.com.

Pyroptosis increases inflammation and induces foam cell formation in atherosclerosis (106). A large portion of cell death in atherosclerotic plaques is attributed to pyroptosis. It also promotes necrotic core formation in advanced atherosclerosis. *In vitro* study shows that low-concentration ox-LDL treatment induces pyroptosis in human monocyte-derived foam cells (107, 108). Absent in melanoma 2 (AIM2)-dependent macrophage pyroptosis exacerbates atherosclerosis as well. AIM2 inflammasome, which is induced by the Janus kinase 2 (Jak2) mutation Jak2V617F, induces the formation of necrotic cores (109). The consequences of foam cells in atherosclerosis including apoptosis, autophagy, necroptosis, and pyroptosis are depicted in Figure 2.

## Regulation of Foam Cells in Atherosclerosis

Since the formation of foam cells is a hallmark in the initial stage of atherosclerosis, tremendous efforts have been made to unveil the regulatory pathways and mechanisms associated with the process. Foam cells are the incorporative effects of lipid uptake, lipid efflux, and cholesterol esterification. The abnormal accumulation of lipids and cholesterol esters in macrophages may demonstrate that cholesterol efflux is insufficient while lipid uptake and esterification are induced (110).

### Cholesterol Uptake

Lipoproteins can be absorbed into macrophages and other cells through different transporters, which is called cholesterol uptake (111). In the 1970s, Brown and Goldstein laboratories demonstrated that modified LDLs were absorbed by macrophages *via* SRs (112). SRs are the major pathway of cholesterol uptake including CD36 (113) and scavenger receptors A (114). CD36 is a member of class B of the SR family. It is identified as a macrophage receptor of oxLDL and mediates its internalization and degradation (115). Apart from mediating lipid uptake, CD36 also modulates the proliferation, migration, and retention in arterial lesions (116). SRs also have different binding ligands of lipoproteins. By using SR-A and CD36 knockout (KO) mice, it has been demonstrated that CD36 and SR-A contribute to 75–90% of the uptake of modified lipoproteins (117). Macrophages also have new types of receptors. For example, lectin-like oxLDL receptor-1 (LOX-1) contributes to this process (118). LOX-1 overexpression significantly aggravates the development of atherosclerotic lesions (118), while LOX-1 knockout in low-density lipoprotein receptor knockout (LDLR^–/–^) mice has smaller atherosclerotic lesions, suggesting that LOX-1 accelerates the formation of foam cells and is proatherogenic (119). By comparing with LOX-1-deficient macrophages, LOX-1 in the normal macrophages accounts for 10% of oxLDL internalization under basal conditions, while this ratio increases by 40% in lysophosphatidylcholine (LPC)-treated macrophages. Efforts have been made to unveil the molecular mechanisms. Sorting nexin 10 (SNX10) plays a critical role in regulating macrophage function and foam cells. Knockout of SNX10 inhibits the function of CD36 in internalizing modified lipoproteins. SNX10 knockout significantly attenuates the progression of atherosclerosis in mice (120). CD146 facilitates the function of cholesterol uptake by CD36. Knockout of CD146 significantly attenuates the lesion areas in high-fat-diet apoE^–/–^ mice (121). Casein kinase 2-interacting protein-1 (CKIP-1) suppresses the foam cell formations. By CKIP-1 deficiency, scavenger receptor LOX-1 is upregulated with faster progression of atherosclerosis (122). Although cholesterol uptake is mediated by CD36, SR-A, and LOX-1, multiple pathways exist, such as phagocytosis and pinocytosis. Current strategies targeting a single pathway of lipid uptake are difficult to inhibit foam cells. More efforts are needed to get a deeper understanding of cholesterol uptake and its function in foam cells.

### Cholesterol Efflux

Multiple pathways mediates the cholesterol transport from the inside cell to the outside, including simple diffusion and transporter-dependent cholesterol efflux (123). In the normal condition, simple diffusion contributes to the majority of cholesterol efflux. In the lipid overload, ABCA1, ABCG1, and scavenger receptors BI (SR-BI) contribute 60–70% of cholesterol efflux (124). Cholesterol in the macrophage foam cells in the body can be transported into high-density lipoprotein (HDL) or apolipoprotein AI (Apo-AI) (125). This process is the initial step of HDL’s reverse cholesterol transport and one of the mechanisms by which it plays a protective role in the cardiovascular system (44). The three major transporters mediating this important process are ABCA1, ABCG1, and SR-BI (126).

ABCA1 transports a variety of substrates out of cellular membranes (127). In cholesterol efflux, ABCA1 facilitates free cholesterol to lipid-poor ApoA-1 and produces nascent HDL (128). Homozygous loss of function mutations in human ABCA1 result in the ultra-low level of HDL cholesterol (129). It has been widely acknowledged that the level of HDL-cholesterol is inversely correlated to cardiovascular risks. However, the effect of ABCA1 on cardiovascular risks and atherosclerosis development is controversial. The impact of losing ABCA1 on the patients of Tangier Disease is variable. Forty-four percent shows increased evidence of cardiovascular disease (CVD). However, four different heterozygous mutations in ABCA1 shows no association with CVD risks. Mutation of ABCA1 in apoE^–/–^ mice has little effect on atherosclerosis (130). Therefore, further studies are needed for the effect of ABCA1 on CVD risks and the development of atherosclerosis (131, 132).

ABCG1 is a transporter of free cholesterol. It functions as a homodimer to transport free cholesterol to HDL particles (133). In humans and mice, the ABCG1 expresses at high levels in multiple organs including the lung, brain, spleen, adrenal glands, heart, and liver (134). Overexpression of ABCG1 leads to a high level of efflux of free cholesterol to HDL particles. (135). Besides, ABCG1 also mediates the efflux of other lipids including oxysterols, phospholipids, and sphingomyelin (136).

### Cholesterol Esterification

Cholesterol esterification is the major process of cholesterol storage in cells. Cholesterol ester is synthesized by acetyl-CoA acetyltransferases (ACAT) at the endoplasmic reticulum (ER) (137). ACAT inhibitors, such as avasimibe and tomatidine inhibit the formation of foam cells and attenuate atherosclerosis in mice (138). However, they fail to attenuate carotid as well as coronary atherosclerosis in patients. Neutral CE hydrolases (NCEH) and hormone-sensitive lipase (HSL) are responsible for the hydrolysis of cholesterol ester in foam cells. NCEH knockout *in vivo* triggers larger plaque areas and more foam cell formation (138). Overexpression of NCEH1 significantly attenuates atherosclerotic lesion areas and induces cholesterol ester hydrolysis and efflux (137). But an NCEH inhibitor is not available and further studies are needed to unveil the mechanism of cholesterol esterification and its consequences on foam cells.

## Molecular Mechanisms of Cholesterol Uptake, Efflux, and Esterification in Foam Cells

**FIGURE 3:**
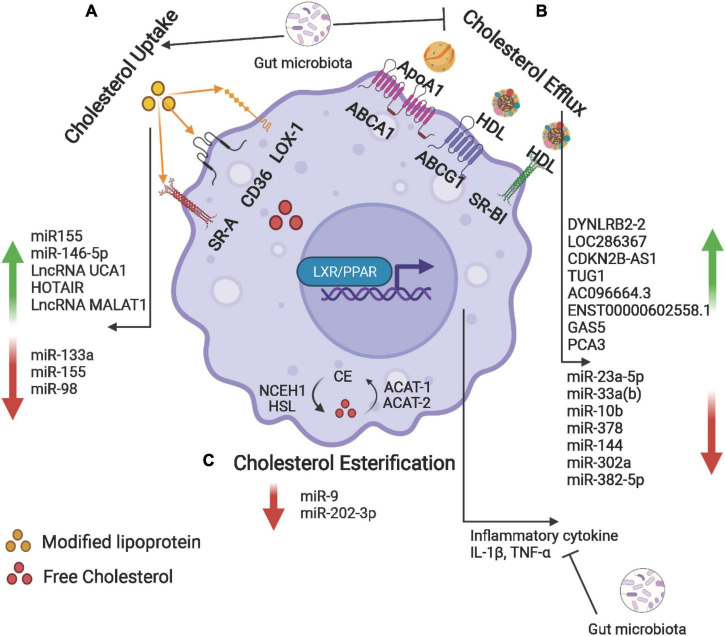
Cholesterol uptake, efflux, and esterification in foam cells. **(A)** Macrophage uptake modified lipoproteins (Ox-LDL) *via* scavenger receptors including SR-A, CD36, and LOX-1. **(B)** Intracellular free cholesterol is efflux to Apo-AI and HDL *via* ABCA1, ABCG1, and SR-BI. **(C)** The internalized free cholesterol is esterified by acetyl-coenzyme A acetyltransferase (ACAT-1 and -2) and stored in lipid droplets. The ester group is removed from cholesteryl by neutral cholesteryl ester hydrolase (NCEH) to release free cholesterol. Non-coding RNAs and gut microbiota may be involved in the regulation of these processes. Created with BioRender.com.

Cholesterol uptake, efflux, and esterification are regulated by multiple nuclear receptors, while non-coding RNAs (ncRNAs) and gut microbiota may also be involved. The process of cholesterol efflux, cholesterol influx and cholesterol esterification, and molecular mechanism in foam cell formation is depicted in Figure 3.

### Nuclear Receptors

The receptors and transporters of cholesterol uptake, efflux, and esterification can be regulated in multiple pathways including transcriptional modulation and post-transcriptional modulation (139). Transcriptional modulation is mediated by a variety of nuclear receptors including liver X receptor (LXR), and PPARα, and PPARγ. LXR is an important nuclear receptor that maintains the hemostasis of intracellular lipids. Synthetic LXR agonists GW3965 and T0901317 can effectively inhibit atherosclerosis in apoE^–/–^ mice and LDLR^–/–^ mice (140). Subsequent studies discover that the mechanism is the induction of ABCA1 and lipid efflux (141). In-depth research on the promoter region of ABCA1 and ABCG1 reveals that the LXR binding site is located in the promoter region of ABCA1 and ABCG1 in humans and mice. However, LXR agonists increase sterol regulatory element-binding protein 1c (SREBP-1c) in the liver, and significantly induce lipid synthesis-related genes including fatty acid synthase (FAS) and acetyl-coenzyme A carboxylase (ACC), resulting in hypertriglyceridemia (142, 143). Selective LXR agonists are developed such as N, N-dimethyl-3-hydroxy-cholenamide (DMHCA), and WAY-252623. These compounds can promote cholesterol efflux from macrophages, and at the same time harm the liver (144). PPARα and PPARγ agonists can significantly induce the level of ABCA1 and cholesterol efflux (141). Mechanism studies reveals that this effect of PPARα and PPARγ agonists is achieved by promoting the expression of LXRα, and further by increasing the expression of LXRα to promote the expression of ABCA1 (145).

### Non-coding RNAs

Different from transcriptional regulation, post-transcriptional regulation focuses on ncRNAs. The past decade has brought a greater understanding of ncRNAs in the regulation of foam cells in atherosclerosis. The function of ncRNAs on the progression of atherosclerosis has been explored (146, 147). NcRNAs are classified as small ncRNAs and long ncRNAs. Small ncRNAs (< 200 bp) include microRNA (miR, miRNA), siRNAs (small interfering RNAs), and piRNAs (PIWI-interacting RNA). ncRNAs, longer than > 200 bp, are called lncRNAs.

MicroRNA (miR, or miRNA) comprise a family of ncRNAs with 22 nucleotides. They can target a 3’ untranslated region of multiple RNAs to induce the degradation of target genes (148, 149). The target gene and the function of miRNAs in the regulation of foam cell formation are listed in Table 1. Single miRNA can be involved in different biological processes. MiRNA mimics or antagonism can also be targeted by different miRNAs, which is a limitation for its application (150).

**TABLE 1:** MicroRNAs in the regulation of foam cells and lipid accumulation.

MicroRNA	Target	Function	References
miR-23a-5p	ABCA1, ABCG1	Inhibiting cholesterol efflux	(151)
miR-10b	ABCA1	Inhibiting cholesterol efflux	(152)
miR-378	ABCG1	Inhibiting cholesterol efflux	(153)
miR-382-5p	ABCA1, ABCG1, CD36	Inhibiting cholesterol efflux	(154)
miR-302a	ABCA1	Inhibiting cholesterol efflux	(155)
miR-144	ABCA1	Inhibiting cholesterol efflux	(156)
miR-33a/b	ABCA1	Inhibiting cholesterol efflux	(157)
miR-155	CD36, HBP1	Inducing lipid uptake and cholesterol efflux	(158)
miR-133a	LDLRAP1	Inhibiting lipid uptake and foam cell formation	(159)
miR-98	LOX-1	Inhibiting lipid uptake	(160)
miR-9	ACAT-1	Inhibiting cholesterol esterification	(161)
miR-202-3p	NCEH-1	Inhibiting cholesterol esterification	(162)

LncRNAs have a size over 200 bp and they can function in both cis and trans regulation of target genes (163, 164). The target gene and the function of lncRNAs in the regulation of foam cell formation are listed in Table 2.

**TABLE 2:** LncRNAs in the regulation of foam cells and lipid accumulation.

MicroRNA	Target	Function	References
DYNLRB2-2	ABCA1, TLR2	Inhibiting inflammation and increasing cholesterol efflux	(165)
LOC286367	ABCA1	Increasing cholesterol efflux	(166)
CDKN2B-AS1	ABCA1	Increasing cholesterol efflux	(167, 168)
TUG1	ABCA1	Increasing cholesterol efflux	(169)
AC096664.3	ABCG1	HDL biogenesis, cholesterol efflux	(170)
ENST00000602558.1	ABCG1	HDL biogenesis, cholesterol efflux	(171)
GAS5	EZH2, ABCA1	Increasing cholesterol efflux	(172)
PCA3	miR-140-5p, ABCA1	Increasing cholesterol efflux	(173)
HOTAIR	miR-330-5p, CD36	Lipid uptake and foam cell formation	(174)
MALAT1	CD36	Lipid uptake and foam cell formation	(175)

NcRNAs have important roles in the regulation of foam cells. Targeting ncRNA is an attractive strategy of therapeutic approaches (8). Antisense oligonucleotides (ASO) target the mature miRNA and lead to the specific miRNA for degradation. Adeno-associated virus (AAV) has been explored to deliver miRNA to a specific organ. Multiple strategies including liposomes, nanoparticles have been explored and developed for the delivery of miRNAs. Furthermore, the stability of miRNA mimics and inhibitors are sufficient to maintain in plasma and can be delivered easily to the gene targets. However, it is challenging for viral and non-viral approaches to deliver miRNA mimics and inhibitors to a specific cell line (176, 177). Although preliminary studies show a positive effect on targeting cells and their gene expression (146, 148), more efforts on delivery systems are needed for clinical trials. Among all of the ncRNAs, miRNA-33 has been studied extensively. The decrease of efflux of cholesterol causes lipid accumulation in cells and accelerates the formation of foam cells. Studies have shown that miRNA-33 can inhibit the expression of ABCA1 and ABCG1, thereby inhibiting cholesterol efflux (178). Antisense oligonucleotides targeting miRNA-33 can effectively boost cholesterol efflux and alleviate the lipid accumulation in macrophage-foam cells *via* increasing the level of ABCA1 and ABCG1 and finally attenuating the atherosclerotic plaques in LDLR^–/–^ mice. Administrations of miRNA-33 ASO for 12 weeks in non-human primates can effectively increase the expression of ABCA1, and further induce the concentration of HDL and reduce the concentration of VLDL triglycerides (179, 180). Clinical trials targeting miRNA have been explored on heart failure and hepatitis C infection (181–183) but miRNAs targeting foam cell formation require clinical evaluation in the future.

### Gut Microbiota

Gut microbiota is a population of microorganisms that colonizes the gastrointestinal tracts. There is large and compelling evidence that the microbiota is involved in atherosclerosis. Several studies focus on the impact of the microbiome-derived metabolites on atherosclerosis including trimethylamine-N-Oxide (TMAO), indoxyl sulfate, and short-chain fatty acid (SCFA). Multiple studies have evaluated the effect of microbiome-derived metabolites on foam cell formation. TMAO induces the level of scavenger receptors and inhibits cholesterol efflux in macrophages leading to the accumulation of foam cells (184). Besides, TMAO is also involved in the migration of macrophages to the plaque areas and induces the secretion of inflammatory cytokines including IL-6 and TNF-α (185). Furthermore, TMAO can also accelerate atherosclerosis by inducing pyroptosis *via* succinate dehydrogenase complex subunit B (SDHB)/ROS signaling pathway (186). Indoxyl sulfate, a byproduct of dietary nutrients, can enhance oxidative stress and inflammation, besides, it also reduces cholesterol efflux and induces foam cell formation (187). Toxic microbiota metabolites, p-cresol has been demonstrated to correlate with the lipid accumulation in macrophages increasing CVD risk (188). Administration of butyrate, a four-carbon SCFA, may alter the species of gut microbiota and induce the cholesterol efflux function in macrophages by the mechanism of up-regulating ABCA1 in high fat diet-fed mice. These results demonstrate the potential of butyrate against atherosclerosis development (189).

Apart from influencing the cholesterol uptake and efflux in macrophages, gut microbiota modulates the foam cells *via* the inflammatory pathway. Since reducing inflammation inhibits the development of atherosclerosis, studies have explored the impact of gut microbiota on inflammation caused by foam cells. SCFAs, including acetate, butyrate, and propionate, are produced by the gut microbiota (190). In the past decade, SCFAs have been playing a key role in the modulation of inflammatory processes of foam cells. In apoE knockout mice, the addition of butyrate in the diet reduces atherosclerosis and lowers the secretion of inflammatory cytokines. Colonic infusions of SCFA in humans (high acetate containing SCFA mixture) significantly alleviate the level of pro-inflammatory cytokine IL-1β (191). Similarly, colonic acetate infusion generates a lower level of TNF-α in humans (192).

## Conclusion and Future Perspectives

Foam cells are the hallmark of the initiation of atherosclerosis. The cell origins of foam cells are not only from monocytes but also from VSMCs, SPCs, and endothelium cells. Novel technologies such as lineage tracing and scRNA-seq have revolutionized our understanding of subtypes of monocytes and VSMCs-derived foam cells. Three main clusters of the macrophages include resident-like, inflammatory, and TREM2*^hi^*. The progression of foam cells leads to diverse processes of programmed cell death including apoptosis, autophagy, necroptosis, and pyroptosis. The formation of foam cells is affected by cholesterol uptake, efflux, and esterification. Novel molecular mechanisms including nuclear receptors, ncRNAs, and gut microbiota are involved in the regulation of foam cells.

Foam cells are an attractive target for treating atherosclerosis. But therapy targeting foam cells is missing for the treatment of atherosclerosis. The reason lies in the inadequate understanding of foam cells. In this review, the novel insights into the origins, consequences, and regulation have been summarized extensively. Since clinical trials of ASOs targeting miRNA have been explored on heart failure and hepatitis C infection (181–183), therapies targeting non-coding RNAs are worth developing and validating in clinical studies.

Nevertheless, the goal of controlling foam cells faces several obstacles. Since the heterogeneity of monocytes and VSMCs, it is difficult to target a specific subtype for therapeutics (11). Second, conclusive results are missing on whether the basal and excessive levels of programmed cell death is beneficial or harmful for controlling the progression of foam cell (43). Therefore, further in-depth research and therapeutic exploration targeting foam cells are needed for the better management of atherosclerosis.

## Author Contributions

YG collected the literature and wrote the manuscript. HZ reviewed the manuscript. RC conceived the idea and reviewed the manuscript. All authors read and approved the final manuscript for publication.

## Conflict of Interest

The authors declare that the research was conducted in the absence of any commercial or financial relationships that could be construed as a potential conflict of interest.

## Publisher’s Note

All claims expressed in this article are solely those of the authors and do not necessarily represent those of their affiliated organizations, or those of the publisher, the editors and the reviewers. Any product that may be evaluated in this article, or claim that may be made by its manufacturer, is not guaranteed or endorsed by the publisher.
